# Effect of high-power probe ultrasonication (20 kHz) treatment duration on the physicochemical, bioactive, mineral and microbiological quality of cranberry (*Vaccinium macrocarpon*) juice

**DOI:** 10.1016/j.ultsonch.2026.108048

**Published:** 2026-09-10

**Authors:** Mabel Kyei Kwofie, Valerie Orsat

**Affiliations:** Bioresource Engineering Department, McGill University, 21111 Lakeshore, Sainte-Anne-de-Bellevue, QC H9X 3V9, Canada

## Abstract

Preservation of bioactive, and nutritional quality alongside microbial inactivation remains a central constraint in cranberry juice production. Thermal pasteurisation, while microbiologically effective, potentially degrades the thermolabile polyphenols fractions that define the functional value of fruit juices. Non-thermal alternatives have been widely reported for polyphenol-rich juices, but these studies almost exclusively report aggregate spectrophotometric endpoints, which cannot distinguish the release of simple phenolics from the degradation of the oligomeric proanthocyanidins that carry cranberry-specific bioactivity. The treatment-duration dependence of A-type versus B-type procyanidins under controlled high-power sonication of cranberry juice has consequently not been resolved, this study addresses that gap. Cranberry juice was treated with 20 kHz probe ultrasonication (SONICS Vibracell VCX 500; 13 mm titanium probe; 60% amplitude; 5 s on/5 s off pulse; at 25 °C for 1, 3, 5 and 7 min on a crushed-ice bath, and the physicochemical, bioactive, mineral and microbiological responses were quantified.

Moderate treatment (3–5 min) increased total flavonoid content by 42.3% and total phenolic content by 33.9% relative to the untreated control, consistent with cavitation-induced disruption of the cell matrix and release of matrix-bound conjugates. Total carotenoid content rose progressively (+116.9% at 7 min), whereas total anthocyanin content, DPPH radical scavenging activity and ferric-reducing antioxidant power declined, indicating that the phenolic pool released by sonication has a lower per-unit radical-scavenging capacity than the anthocyanin and oligomeric fraction it displaces. Procyanidin A1 was stable to 5 min (within 3.4% of the control at every duration to 5 min, and within 1.1% at 3 and 5 min) while procyanidin B2 fell progressively to 20.2% below the control at 7 min, a structure-dependent divergence attributable to the additional C-O-C inter-flavan bond of A-type dimers. A 2.00 log CFU/mL (99%) reduction in total plate count of inoculated *Escherichia coli K12* was achieved at 3 min, with no further measurable reduction to 7 min. Treatment for 3 min therefore represents the optimum, delivering the maximum microbial reduction observed at the point of greatest phenolic and flavonoid release and before any measurable loss of procyanidin A1. These results define a duration window for ultrasonication of cranberry juice and provide the procyanidin-resolved basis required for scale-up of the process to continuous-flow industrial configurations, mostly as a component of a combined process rather than as a standalone replacement for thermal pasteurisation.

## 1 Introduction

**Fig. 1:**
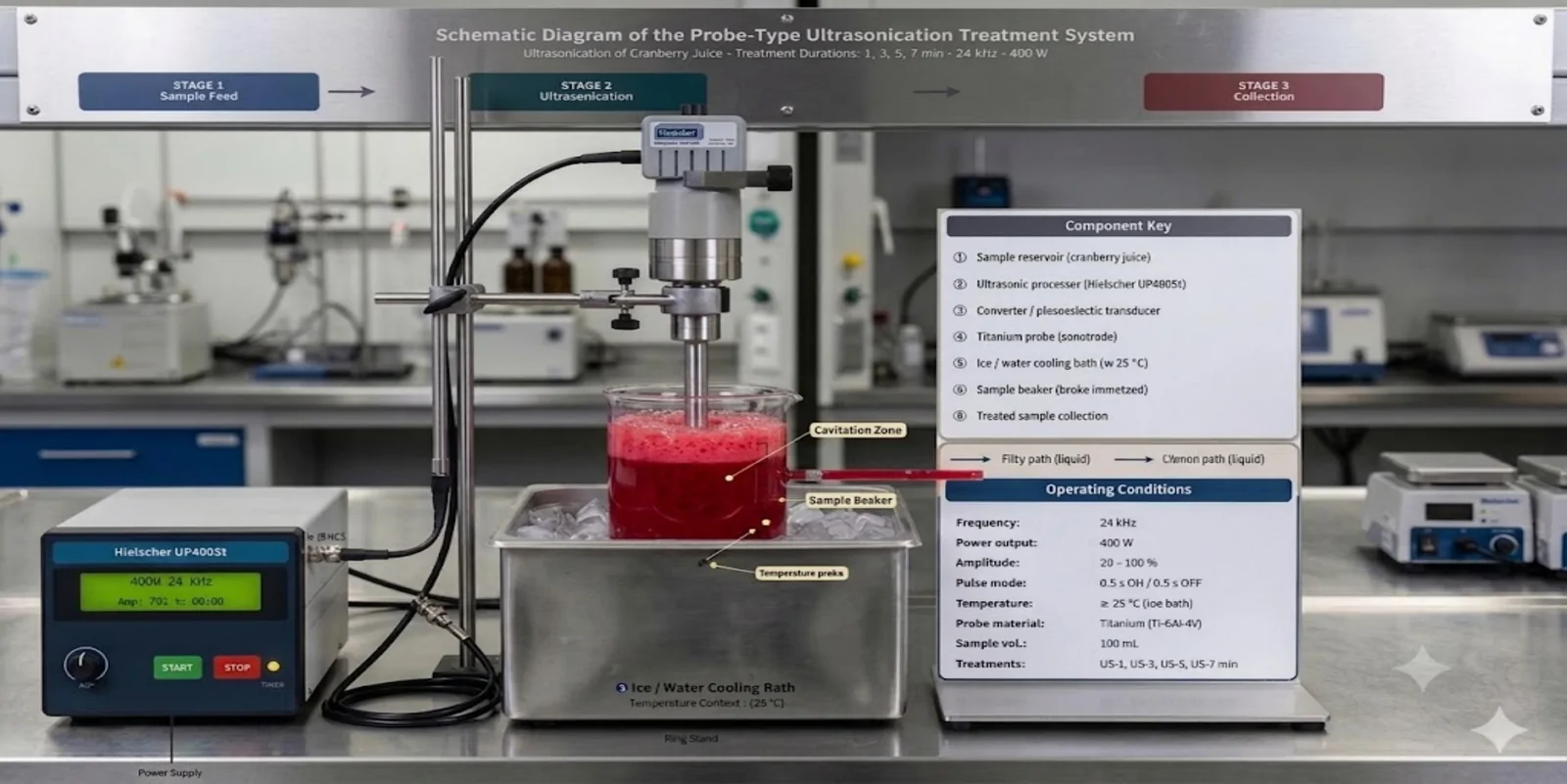
Schematic diagram of the probe-type ultrasonication treatment system.

The food industry has given importance to the development of non-thermal food processing technologies in response to rising customer demand for “fresh-like” produce that keep their natural, nutritional and sensory qualities. Cranberry (*Vaccinium macrocarpon*) juice is particularly valued for its high concentration of bioactive compounds such as anthocyanins, proanthocyanidins, and various phenolic acids, which contribute to its potent antioxidant properties and associated health benefits [1], [2]. Traditional thermal pasteurization, while effective in ensuring microbial safety, frequently reduces these thermolabile compounds, resulting in decreased nutritional value and altered sensory characteristics such as color and flavour [3], [4]. Ultrasonication, using high-power (HPU; 20–100 kHz, 10–1,000 W, generating acoustic intensities > 1 W/cm^2^) ultrasound at frequencies of 20 (kHz) to 100 (kHz), has emerged as a viable “green” potential tool for fruit juice processing [5]. Ultrasound has diverse applications in food processing because of its capacity to produce chemical, physical, and mechanical modifications in food products. The efficiency of ultrasound in liquid media is mostly due to the phenomena of acoustic cavitation, which is the development, expansion, and violent collapse of tiny bubbles created within localized areas at localized temperature up to ∼5000 K and pressure up to ∼500 Mpa transiently within collapsing cavitation bubbles [3]. The mechanical shear pressures and micro-jets generated during bubble collapse destroy microbial cellular walls, allowing intracellular compounds to be released from the plant/food matrixes [4]. Research studies suggest that ultrasonication generally retains fruit juice's essential physicochemical properties [6]. Ultrasound treatments may suppress browning enzymes such as polyphenol oxidase (PPO), preserving the rich red color of cranberry products like cranberry juice [[7], [9], [10], [8]]. Ultrasonication can improve the concentration of bioactive compounds through its food matrixes breakdown hence enhancing extraction properties [[11], [12], [13]]. Sonication has been demonstrated to boost anthocyanin concentration in juices by breaking down cell structures and enhancing pigment accessibility [[2], [14], [15]]. Ultrasound treatment has also been shown to improve the preservation of overall phenolic content [[16], [17], [18]]. To achieve safety standards, foodborne bacteria are frequently reduced by processing by 5 logarithmic units [[19], [20]]. Ultrasound has been used in the reduction of microbial load of yeasts, molds, and aerobic bacteria [[21], [22], [23]]. Despite the benefits of ultrasonication, its influence varies greatly depending on the individual processing parameters, such as amplitude, time, and temperature [[24], [25], [26]]. Optimizing these characteristics is critical for cranberry juice, which has a high acidity and a distinct polyphenolic profile, to improve nutritional retention while maintaining commercial safety [[27], [28], [29]]. This study seeks to evaluate the impacts of varied ultrasonic durations and parameters on the qualitative, microbiological, bioactive and nutritional properties of processed cranberry juice.Fig. 1.

The physical basis of ultrasonic processing is acoustic cavitation, and the two regimes in which cavitation operates have opposite consequences for juice quality. When a sound wave of sufficient pressure amplitude propagates through a liquid, the rarefaction half-cycle lowers the local pressure below the tensile strength of the medium and pre-existing gas nuclei grow into cavitation bubbles. Bubbles that oscillate about an equilibrium radius over many cycles (stable cavitation) generate microstreaming and sustained shear at the bubble wall. Bubbles that grow beyond a resonance threshold and collapse within a single compression half-cycle (transient cavitation) implode near-adiabatically, producing the localised hot spots of approximately 5000 K and 500 MPa, together with asymmetric micro-jets that impinge on nearby surfaces at velocities of the order of 100 m s-1. The mechanical regime, comprising shear, micro-jetting and micro-turbulence, dominates matrix disruption and therefore governs the release of cell-wall-bound phenolics, carotenoid-protein complexes and intracellular minerals; it is also the principal route to microbial cell lysis. The chemical regime, comprising sonolysis of water within and around the collapsing bubble to yield hydroxyl radicals and hydrogen atoms and, on recombination, hydrogen peroxide, dominates oxidative degradation and therefore governs the loss of anthocyanins, ascorbic acid and B-type procyanidins. Both regimes scale with delivered acoustic energy, so the net effect of ultrasonication on any juice is a competition between extraction and degradation whose balance shifts with treatment duration. This competition, rather than any single monotonic mechanism, is the framework within which the results of this study are interpreted. The sonochemical behaviour of cavitating systems has been characterised in detail in the wider sonochemistry literature, where the same cavitation events are exploited deliberately for radical generation, water sonolysis and sonochemical hydrogen production, and where the dependence of radical yield on frequency, acoustic power, dissolved gas composition, liquid temperature and reactor geometry has been systematically mapped [[30], [31], [32], [33]]. Two findings have been noted. first, hydroxyl radical production per unit delivered energy is strongly non-linear in acoustic power, so that modest changes in amplitude or duty cycle can shift a treatment from a predominantly mechanical to a predominantly oxidative regime; this is why treatment duration and delivered energy density, rather than nominal generator rating, are the parameters reported throughout this work. Second, radicals generated in the bubble interior escape into the bulk liquid only over short diffusion distances, so oxidative damage in a real food matrix is spatially heterogeneous and is buffered by the antioxidant capacity of the medium itself. Both findings are included in the interpretation of the ascorbic acid and anthocyanin data in this study.

The recent literature on ultrasonication of polyphenol-rich juices is substantial but converges on a narrow set of endpoints. Studies published within the last five years have overwhelmingly reported total phenolic content, total flavonoid content, total anthocyanin content and one or two antioxidant assays, with the near-universal finding that short sonication raises the aggregate phenolic pool while anthocyanins decline [[3], [34], [35]]. These aggregate spectrophotometric endpoints are informative about extraction efficiency but are structurally limited. For cranberry this distinction is decisive. The health claim that differentiates cranberry from other red fruits rests on A-type proanthocyanidins, whose double C-O-C and C-C inter-flavan linkage confers anti-adhesion activity against P-fimbriated uropathogenic *Escherichia coli* that B-type dimers do not possess [[14], [27]]. Whether high-power ultrasonication preserves this A-type fraction, and whether it does so over the same duration window that achieves microbial reduction, has not been established. Gomes et al. [2] sonicated cranberry juice but reported proanthocyanidins in aggregate without resolving linkage type, and combined ultrasound with high-pressure processing so that the ultrasonic contribution cannot be isolated; no subsequent study has reported compound-specific A-type versus B-type quantification across a sonication time course [14].

The present study addresses this gap by combining LC-ESI-Q-TOF-MS/MS quantification of procyanidin A1 and procyanidin B2 with a full physicochemical, mineral, antioxidant and microbiological panel measured on a single set of samples, so that the duration at which microbial reduction is achieved can be placed directly against the duration at which the cranberry-specific bioactive profile begins to degrade. The specific objectives were to (i) quantify the effect of 20 kHz probe ultrasonication duration on the physicochemical stability of cranberry juice; (ii) resolve the divergent responses of the aggregate phenolic pool, the anthocyanin fraction and the individual A-type and B-type procyanidins; (iii) determine the ICP-OES-detectable mineral profile as a chemically inert tracer of matrix disruption; (iv) establish the microbial inactivation achieved against inoculated *E. coli K12*; and (v) identify the treatment duration that best reconciles these competing responses.

## 2 Materials and methods

### 2.1 Materials, reagents, solvents and standards

Frozen cranberries obtained from the Super C grocery store in Salaberry-de-Valleyfield, Quebec, Canada, were used for this research study. The cranberries were rinsed with distilled water and stored in a refrigerator to defrost. The following materials were procured from Sigma, Canada: Folin-Ciocalteu reagent, glacial acetic acid, sodium acetate trihydrate, 2,4,6-tripyridyl-s-triazine (TPTZ), hydrogen chloride (HCl), ferric chloride, Trolox, potassium chloride, methanol, sodium nitrate, aluminum chloride, sodium hydroxide, quercetin/catechin standard, sodium carbonate, gallic acid standards, ethanol, acetone, HPLC vials with caps, sterile polystyrene petri dishes, sodium chloride, yeast extract, bacteriological peptone, and agar.

### 2.2 Preparation of the cranberry beverage

The washed and defrosted cranberries were processed in a domestic food processor for 20 min at ambient laboratory temperature (22 ± 2 °C), and the resulting slurry was vacuum filtered through a cheese cloth to yield the cranberry juice used for all experiments. The filtered juice was dispensed into sterile amber glass bottles with the headspace minimised to limit oxidative change and stored at −18 °C for no longer than four weeks before use. A single homogeneous juice batch was prepared and used for all treatments and for the untreated control, so that every comparison reported in the manuscript is within-batch. Frozen aliquots were thawed overnight at 4 °C and equilibrated to 24 °C immediately before treatment. All analyses were performed on the day of treatment, with samples held at 4 °C in the dark for no more than 4 h between sonication and analysis.

### 2.3 Ultrasonication unit set-up and processing

Ultrasonication was performed with a 20 kHz probe ultrasonic processor (SONICS Vibra-Cell (TM) VCX 500, 500 W nominal generator rating; Newtown, Connecticut, USA) fitted with a 13 mm diameter solid titanium-alloy probe of tip cross-sectional area 1.33 cm2. For each run, 100 mL of thawed juice equilibrated to 24 °C was transferred to a sterile 250 mL glass beaker, and the probe was centred in the beaker and immersed to a depth of 25 mm below the liquid surface. Treatments were applied at 60% amplitude in pulsed mode with a 5 s on/5 s off duty cycle, for total elapsed treatment times of 1, 3, 5 and 7 min. At this setting the instantaneous delivered power during the ON pulse was approximately 300 W, corresponding to an instantaneous acoustic intensity of approximately 225 W cm-2 at the probe tip. Because the probe is energised for half of the elapsed time, the time-averaged delivered power over the treatment period was approximately 150 W, and the total delivered acoustic energy was therefore approximately 9,000, 27,000, 45,000 and 63,000 J for the 1, 3, 5 and 7 min treatments respectively. The generator energy cut-off was set to 120,000 J, above the energy delivered in any treatment, so that every run terminated on elapsed time rather than on the energy limit.

Throughout treatment the beaker was seated in a crushed-ice bath maintained above the liquid level of the sample, and the ventilation ports of the VCX 500 were kept unobstructed to prevent transducer overheating. Sample temperature was measured with a digital thermocouple probe immediately before and immediately after each treatment, and the post-treatment temperature did not exceed 25 °C in any run. It should be stated explicitly that a continuous temperature profile was not logged during sonication. The present data therefore do not exclude transient localised heating within the cavitation zone, which is intrinsic to the process and cannot be eliminated by external cooling. The treatment is accordingly described here as temperature-controlled rather than strictly isothermal, and the term non-thermal is used in its conventional processing sense, namely that no external thermal energy is applied and the bulk sample is held below the threshold at which thermal degradation of anthocyanins and ascorbic acid becomes significant, rather than as a claim of constant temperature.

### 2.4 Rationale for the selection of characterisation methods

The analytical panel was assembled to resolve the two competing consequences of cavitation described in the Introduction, rather than to catalogue quality attributes. Total soluble solids, pH and CIE L*a*b* colorimetry test whether the treatment is compositionally and visually conservative at the bulk level, and therefore whether the product remains acceptable. Folin-Ciocalteu total phenolics and aluminium chloride total flavonoids measure the aggregate extractable pool and are read here as indicators of mechanical matrix disruption. The pH-differential total anthocyanin assay and the DPPH and FRAP assays are read as indicators of the oxidative regime, since the flavylium chromophore and the single-electron-transfer capacity of the juice are the endpoints most sensitive to hydroxyl radical attack. LC-ESI-Q-TOF-MS/MS quantification of procyanidin A1 and B2 supplies the structural resolution that the spectrophotometric assays cannot, allowing the stability of the doubly linked A-type dimer to be compared directly with that of the singly linked B-type dimer under identical acoustic conditions, which is the central structure–property relationship of this study. ICP-OES mineral profiling provides an independent and chemically inert tracer of matrix disruption: minerals are neither created nor destroyed by cavitation, so a change in ICP-OES-detectable concentration isolates the extraction term from the degradation term that confounds every organic endpoint. Ascorbic acid and total carotenoids contrast a highly oxidizable hydrophilic vitamin with a lipophilic pigment class sequestered in protein and lipid complexes, and so separate solubility-driven from oxidation-driven responses. Total plate count of inoculated *E. coli K12* establishes whether the duration required for matrix disruption coincides with that required for microbial reduction. Also, the presence of *e. coli* in a food product or liquid medium is an indicator of fecal pollution as well as the presence of other pathogenic bacteria, hence was used in his study [36].

### 2.5 Color, pH, and total soluble solids (TSS)

Color change measurement before and after the application of the ultrasound treatment were done using a Chromameter (Model- CR300, Konica-Minolta, USA), in terms of L* − lightness, a* − redness, and b* − yellowness values. Prior to the tests, the colorimeter was calibrated using a white reference tile (L* − 97.36, a* − 0.09, and b* − 2.81). The pH of the cranberry juice was verified before and after treatment using a buffer solution and a pH probe. A digital refractometer (ATAGO, model PR32a, Tokyo, Japan) was used for measuring the total soluble solids in the untreated and treated cranberry juice samples.

### 2.6 LC-MS quantification of procyanidin A and B

The ultrasonicated cranberry juice samples were filtered using a 0.20 µm PTFE syringe filter (Fisher Scientific) prior to mass spectrometry analysis. Procyanidin A1 (≥95% purity, Sigma-Aldrich, Cat. No. 75513) and Procyanidin B2 (≥90% purity, Sigma-Aldrich, Cat. No. 92350) were used as external standards to construct calibration curves. Quantification was performed separately for each compound. Results are expressed in μg/mL for each procyanidin type individually. The LC-MS analyses were conducted using a LC–ESI–Q–TOF–MS/MS to determine the procyanidin A and B profiles of the treated cranberry samples as well as the effect of the ultrasonication on these bioactive compounds on the cranberry juices’ samples.

### 2.7 Mineral content analysis

Minerals are essential nutritional components of fruit juices and serve as markers of overall juice quality. Ultrasonication-induced acoustic cavitation can alter cell matrix integrity, potentially affecting the release and detectable concentration of minerals from cranberry tissue into the juice matrix. Assessment of macro- and micro-mineral profiles is therefore important for evaluating the nutritional impact of the treatment. The concentrations of potassium (K), calcium (Ca), magnesium (Mg), phosphorus (P), sulphur (S), manganese (Mn), and iron (Fe) and micro-minerals were assessed. The juice samples were digested and subsequently subjected to inductively coupled-plasma optical emission spectrometry (ICP-OES) analysis, as per the methodology outlined by [37], with modifications, since juice was used in this study. Approximately 5 mL samples of cranberry juice are measured into DigiPREP tubes, to which 3 mL of concentrated TraceMetal grade Nitric Acid (68%) and 2 mL of hydrogen peroxide (30%) is added. This is subjected to a DigiPREP block heater at 95 °C for 2 h to achieve complete digestion. After digestion, the transparent and colorless solution is transferred to a 50 mL volumetric flask and adjusted to the calibration mark using deionized water. Ultimately, ICP-OES (Thermo iCAP-6500 series Duo) is employed for the analysis.

Instrument calibration was performed with multi-element standards traceable to NIST, and calibration curves were accepted at R^2^ greater than or equal to 0.999. Reagent blanks carried through the complete digestion procedure were analysed with every batch and blank-subtracted from sample intensities. Method detection limits were established as three times the standard deviation of ten replicate reagent blanks. Copper returned net emission intensities below those of the reagent blank in all samples, including the untreated control, yielding negative blank-corrected concentrations that are not physically interpretable; copper was therefore below the method detection limit under the digestion and dilution conditions used, and it has been removed from Table 3 rather than reported as a numerical value. All reported concentrations are expressed on an as-digested basis and represent the ICP-OES-detectable fraction of each element under the stated protocol. They are not corrected for matrix suppression, and they are intended for comparison between treatments within this study rather than for absolute comparison against compositional values obtained by different digestion or dilution methods.

### 2.8 Ascorbic acid Determination

Spectrophotometric method was used in determining the ascorbic content of the ultrasonicated treated cranberry samples following the approach proposed by [38] with modifications. Briefly, Ferric chloride (FeC1_3_) and 1,10-Phenanthroline solutions are combined with distilled water and the cranberry juice samples, stirred together until dark color change is achieved and absorbance measured at 430 nm. A calibration curve was constructed using L-ascorbic acid standard solutions (0, 10, 25, 50, 75, and 100 mg/L, R^2^ > 0.99), and results are expressed as mg ascorbic acid per liter (mg/L) of juice [38].

### 2.9 Total carotenoids analysis

For the analysis of total carotenoids, 5 ml of cranberry juice samples are mixed with 0.8 ml of 80% (v/v) ethanol in a 10 ml tube and heated to 92 °C for 30 min. The resulting hot ethanol extract is transferred into 2-ml micro test tubes. To the remaining cranberry juice extract, 0.5 ml of 80% (v/v) ethanol is added and allowed to stand for 15 min at room temperature, after which the mixture is vortexed. Subsequently, 0.4 ml of the extract is combined with 5 ml of 80% (v/v) acetone, and absorbance is determined at 663, 646, and 470 nm using a Synergy H4 hybrid microplate reader. The quantification of carotenoids is conducted in accordance with [[39], [40]] with equation (1):

(1) $$\text{Cx}+\text{c}\;=\left(1000{\text{A}}_{470}-3.27\text{Ca}\;-104\text{Cb}\right)/229$$

where C_x+c_ is the Carotenoid content.

$${\text{C}}_{\text{a}}=12.21{\text{A}}_{663}-2.81{\text{A}}_{646};{\;\text{C}}_{\text{b}}=20.13{\text{A}}_{646}-5.03{\text{A}}_{663}$$

The Wellburn [40] trichromatic equations use mathematically established spectrophotometric coefficients derived from pure pigment standards; no separate external standard curve is required for this method.

### 2.10 Antioxidant activity measurement

Antioxidant activity following various non-thermal treatments is assessed using the FRAP assay and DPPH radical scavenging test. The DPPH radical scavenging activity of the samples is assessed using the methodology outlined by [41]. For this study, the sample extract is combined with distilled water as a blank and freshly prepared 0.1 mM 2,2-diphenyl-1-picrylhydrazyl (DPPH). The mixture is incubated for 20 min in darkness, and the absorbance is measured at 517 nm. The free radicals neutralized by DPPH radicals are represented as inhibition percentage and estimated using equation (2).

(2) $$Inhibition\left(\%\right)=\frac{AbsControl\;@\;517\;nm\;-\;AbsSample\;@\;517\;nm}{AbsControl\;@\;517\;nm}$$

The ferric-reducing antioxidant power (FRAP) analysis for the samples is prepared following the methodology outlined by [42]. To summarize, the FRAP reagent is freshly made by combining acetate buffer (pH 3.6), ferric chloride solution, and 2,4,6 tripyridyl-s-triazine (TPTZ) solution. The FRAP solution is activated at 37 °C in a water bath, then Trolox is used as the standard, while distilled water serves as the blank. For the experiment, freshly made FRAP reagents is combined with standard Trolox solution, sample extract, or blank. The mixture is then subsequently incubated in darkness for 30 min, after which the absorbance is measured at 593 nm. The antioxidant capacity of the samples is quantified as FRAP value in micromoles of Trolox equivalents per gram of sample (mmol TE/g) [42].

### 2.11 Total flavonoids content measurement

The total flavonoid concentration of the ultrasonicated cranberry juice sample is assessed using the aluminum chloride colorimetric technique as outlined by [43], with a few modifications. The assay consists of combining the treated samples with distilled water followed by the addition of 5% sodium nitrite. After allowing the sample to stand for 5 min, 10% aluminum chloride is added, and the samples are permitted to react for a further 5 min. Subsequently, 1 M sodium hydroxide and distilled water are incorporated into the samples. The absorbance is measured at 510 nm and compared to a Quercetin standard curve to determine the total flavonoid content in the samples and expressed as mg quercetin equivalent per mL of juice (mg QE/mL); results may also be expressed on a fresh weight basis (mg QE/g FW) using juice density (∼1.04 g/mL) [43].

### 2.12 Total phenolic content measurement

The total phenolic content of the treated cranberry samples is assessed using the Folin–Ciocalteu technique proposed by [44]. The sample is combined with distilled water for the blank and Folin–Ciocalteu reagent for the experimental sample. The mixture is left to react for 5 min, after which a 7.5% sodium carbonate solution is added, and the mixture is incubated in the dark at room temperature for 90 min. Following incubation, absorbance is assessed at 765 nm, and the total phenolic content in the sample is compared with the gallic acid standard curve, and reported as mg gallic acid equivalent (GAE) per gram of fresh weight sample [44].

### 2.13 Microbial loads analysis

The microbial loads were enumerated using the total plate count approach. 100 ml of cranberry juice was inoculated with 1 mL of an overnight culture of *Escherichia coli* K12 strain (ATCC 15597; C-3000) in EC broth (Oxoid, Canada) (approximately 10^7^ cells /ml) and then treated with ultrasonication treatments to determine how effectively the non-thermal technology can decrease the microbial contents. The juice samples (1 ml) were serially diluted in 9 ml saline water by three times, then 1 ml plated on nutrient agar plates and incubated for 24 h then surviving microbes were enumerated. The concentration of bacterial loads is calculated using equation (3):

(3) $$CFU/mL=\frac{Number\;of\;Colonies\;\times \;Dilution\;Factor}{Volume\;of\;Plated\;(mL)}$$

### 2.14 Statistical analysis

The experiment was arranged as a one-factor design with treatment duration (untreated control, 1, 3, 5 and 7 min) as the single fixed factor at five levels. Each treatment level was prepared as three independent replicate sonication runs drawn from the same juice batch on separate days (n = 3 independent replicates), and each replicate was measured in analytical triplicate for the spectrophotometric assays; microbiological counts were performed in triplicate. Data are reported as mean ± standard deviation of the independent replicates. Data were then subjected to one-way analysis of variance (ANOVA), and where the treatment effect was significant the means were separated using the Tukey honestly significant difference (HSD) post-hoc test at a family-wise significance level of p < 0.05; treatments not sharing a common superscript letter within a column or figure differ significantly. Since each response variable was analysed by a separate one-way ANOVA, the reported significance levels are not corrected for multiplicity across response variables, and comparisons made across variables should be interpreted as such. All analyses were performed in OriginPro 2024 (OriginLab Corporation, Northampton, MA, USA).

## 3 Results and discussion

### 3.1 Analysis of the variation in TSS, pH and colour of treated cranberry juice

The effects of ultrasonication at treatment durations of 1, 3, 5, and 7 min on the physicochemical quality parameters of cranberry juice are summarized in Table 1. These parameters provide a first-level assessment of the physical and chemical stability of the juice following non-thermal treatment.

**Table1:** Effect of ultrasound treatment on some physicochemical properties of cranberry juice.

Sr No.	Sample	TSS (°Brix)	pH	L*	a*	b*	ΔE	
1	Untreated	9.80 ± 0.73^a^	2.60 ± 0.16^a^	21.15 ± 0.07^a^	10.47 ± 0.06^d^	5.25 ± 0.18^b^	0.00^e^	
2	US 1 min	9.98 ± 0.78^a^	2.53 ± 0.18^a^	19.83 ± 0.21^d^	10.96 ± 0.11^bc^	5.75 ± 0.02^a^	1.50^b^	
3	US 3 min	9.75 ± 0.97^a^	2.49 ± 0.14^a^	19.46 ± 0.04^e^	11.14 ± 0.01^b^	5.12 ± 0.01^b^	1.82^a^	
4	US 5 min	9.65 ± 1.17^a^	2.51 ± 0.14^a^	20.32 ± 0.01^c^	10.74 ± 0.02^cd^	5.33 ± 0.02^b^	0.88^d^	
5	US 7 min	9.78 ± 1.18^a^	2.50 ± 0.17^a^	20.67 ± 0.04^b^	11.55 ± 0.01^a^	4.75 ± 0.04^c^	1.29^c^	

The physicochemical parameters of cranberry juice subjected to ultrasonication for varying durations (1, 3, 5, and 7 min) are presented in Table 1. Total soluble solids (TSS) ranged from 9.80 ± 0.73 brix of the untreated control to 9.78 ± 1.18 for (US-7), 9.65 ± 1.17 brix at 5 min (US-5), 9.75 ± 0.97 (US-3) through to 9.98 ± 0.78 (US-1). One way ANOVA showed no significant difference, while Tukey HSD also showed that all results were statistically equivalent. A minor decline in the total solids could have occurred with the ultrasonication treatments attributable to induced cavitation potentially degrading some of soluble carbohydrates and the distraction of solute structures of macromolecules, which can diminish the refractive index of the liquified solid elements [2], however this was not the case for this study. Similar research studies of fruit juices processing reported decreases of total solids (TSS) with ultrasonication treatments [34] as they are connected with shear forces created by acoustic cavitation leading to disruption of pectin and hemicellulose.

Quantitatively, the complete absence of a TSS effect here (9.65–9.98 degrees brix, p > 0.05, maximum deviation from the control 1.8%) contrasts with the 3–7% reductions reported for phalsa juice at comparable amplitude [34], and with the reduction reported for cranberry juice by [2]. The difference is attributable to matrix composition rather than to treatment intensity. The juices in which TSS falls are those carrying an appreciable pectin and hemicellulose load, whose cavitational depolymerization lowers the refractive index of the soluble fraction. The vacuum-filtered cranberry juice used here was largely depleted of suspended pectic material before treatment, leaving simple sugars and organic acids that are not susceptible to cavitational shear at the energies applied. The practical implication is that TSS is not a sensitive indicator of cavitation dose in a clarified juice.

The pH of the ultrasonicated samples remained stable across all treatments, ranging from 2.49 ± 0.14 (US-1) to 2.60 ± 0.16 (untreated control), and ANOVA revealed no significant treatment effect (p > 0.05). Sonication of aqueous media can in principle lower pH through the formation of weak acids from dissolved gases and through oxidative breakdown of carbohydrates during cavitation collapse [4] but no such acidification was measurable here. The explanation most consistent with the data is shielding, cranberry juice carries a high concentration of quinic, malic and citric acids and is already at pH near 2.5, so the quantity of acid generated sonochemically at the energies applied is negligible against the existing acid load, and the total observed change of 0.11 pH units lies within the replicate standard deviation. This is offered as the most probable interpretation rather than as a demonstrated mechanism. The practical consequence of pH stability is that the acid-dependent equilibrium of the anthocyanin flavylium cation is preserved, so any anthocyanin loss measured below can be attributed to degradation rather than to a shift in the colour equilibrium [45].

Color changes were evaluated using CIE L*a*b* coordinates and the total color difference (ΔE). The L* values ranged from the control sample 21.15 ± 0.07, being the highest, and the US-3 with19.46 ± 0.04 having the lowest L*. A darkening effect was observed with treatment US-1 (19.83) and US-3 (19.43) which were the shorter duration treated samples, with a partial recovery with US-5 (20.32) and US7 (20.67) which were longer duration treated samples. A slight decrease in L* (lightness) from 21.15 ± 0.07 in the control to 19.46 ± 0.04 with US-3 suggesting marginal darkening, may reflect anthocyanin polymerisation or the release of intracellular pigments (anthocyanins, flavonoids) under acoustic shear forces [[9], [10]]. Tukey HSD test found the treated samples distinct.

The a* (redness) values indicate an increase in US-7 min ultrasonicated sample with 11.55 ± 0.01, higher than the control sample with 10.47 ± 0.06. The intermediate duration treated samples revealed a non-linear trend: US-3 had 11.14 ± 0.1, US-1 with 10.96 ± 0.11, and US-5 having 10.74 ± 0.02. Increase in redness could be attributed to the release of anthocyanin from disrupted cell matrices, however such a conclusion could not be drawn here. Studies conducted by Ordóñez-Santos et al. [18], also reported of minimal ΔE alterations with ultrasonicated juice samples, establishing that ultrasonication treatments may not substantially affect the visual quality of juice processed at relatively short treatment durations [18]. All the samples had distinct Tukey HSD groups. Although one-way ANOVA and Tukey HSD analysis confirmed statistically significant differences in ΔE among treatments (p < 0.05), all ΔE values fell within the perceptually minimal range (1 < ΔE < 3), indicating differences below the threshold of human visual detection. Statistical groupings are provided in Table 1.

The b* (yellowness) values of the untreated control sample registered at 5.25 ± 0.18, which is similar in values with most ultrasonic treatments with US-3 at 5.12 ± 0.01, US-5 at 5.33 ± 0.02, and US-7 revealing the lowest value of 4.75 ± 0.04 indicating statistical significance. Also, US-1 had the highest value of 5.75 ± 0.02 suggesting elevated yellowness with shorter treatment durations and reduced yellowness with longer treatments. The elevated b* at US-1 most likely reflects acoustic cavitation-induced release of yellow-pigmented flavonols (e.g., quercetin and myricetin glycosides) from disrupted cell walls, while the progressive decline in b* at US-7 is attributable to oxidative degradation of these chromophores by hydroxyl radicals generated during sustained cavitation collapse. Overall, the colour change observed with the ultrasound treatment of the cranberry juice, was minor (1 < ΔE < 3) and below the threshold which would be considered as a visually objectionable alteration with food products.

### 3.2 Total phenolic content, total flavonoid content, total anthocyanin content, DPPH radical scavenging activity, and FRAP analysis

**Fig. 2:**
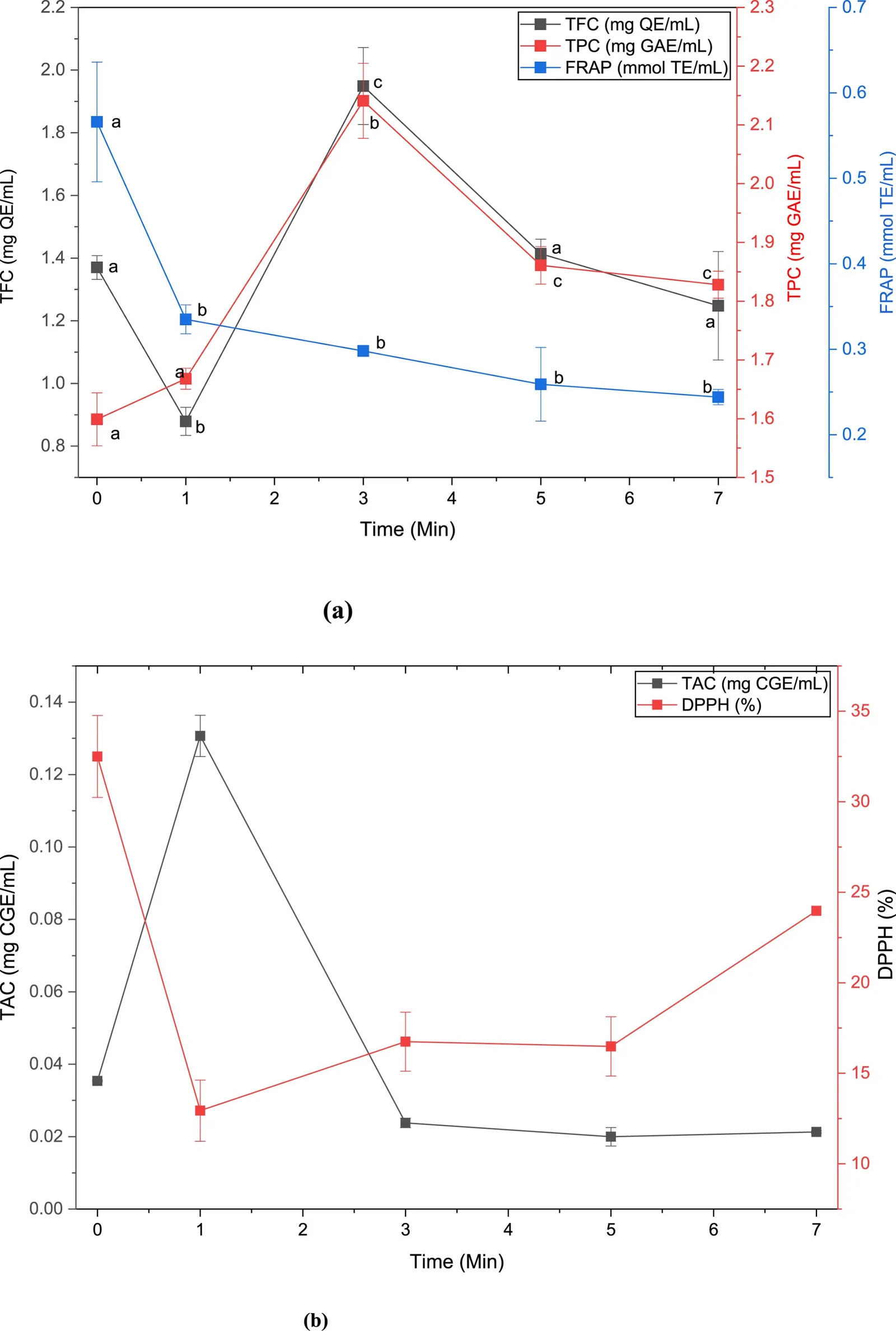
Effect of ultrasonication treatment on (a) total flavonoids (TFC), total phenolics (TPC), and ferric-reducing antioxidant power (FRAP) and (b) total anthocyanins (TAC), DPPH radical scavenging activity of cranberry juice. Statistical difference is indicated with a different letter (a, b or c).

The effects of ultrasonication at varying time durations of 1, 3, 5, and 7 min on the spectrophotometric bioactive parameters of cranberry juice samples are presented in Fig. 6.2. The cranberry sample’s total phenolic content (TPC) showed a non-monotonous pattern with treatment times. The ultrasonicated juice of 1 min had the lowest total phenolic content value of 0.668 ± 0.018 mg GAE/mL, which is 58.2% decrease from the control sample with 1.599 ± 0.045 mg GAE/mL. This initial reduction reflects the quick impact on phenolics from the intense acoustic cavitation at minimal treatment durations, before the subsequent dominance of acoustic cell wall breakdown [46]. Statistical significance of all reported differences was determined by one-way ANOVA and Tukey HSD post-hoc test (p < 0.05); treatment groups sharing the same letter in Fig. 2 are not significantly different.

Ultrasonicated samples of 3 min had a high total phenolic content (TPC) of 2.141 ± 0.064 mg GAE/mL, exceeding that of the control by 33.9%. The increase is attributable to cavitation-induced breakdown of plant cellular walls and the hydrolysis of matrix-bound glycosidic bonds, hence the release of conjugated phenolics that were previously inaccessible to the Folin–Ciocalteu reagent [47]. At US-5 and US-7, the total phenolic contents (TPC) of the ultrasonicated juices following 5 and 7 min treatments declined to 1.861 and 1.828 mg GAE/mL respectively, which suggests that extended ultrasonication may have promoted oxidative degradation of the released phenolic compounds [48], while the treated juices still had an increase in TPC over the control.

The total phenolic content trajectory, a fall to 0.668 ± 0.018 mg GAE/mL at 1 min, 58.2% below the untreated control of 1.599 mg GAE/mL, followed by a rise to 2.141 ± 0.064 mg GAE/mL at 3 min, is an uneven feature of this dataset. Two interpretations are tenable. The first is physical, at 1 min the cavitation field has disrupted cell fragments sufficiently to release proteins and pectic fragments that complex with phenolics and mask them from the Folin-Ciocalteu reagent, and sufficiently to generate transient particulate that is removed at the filtration step preceding the assay, so that the 1 min sample is masked and depleted rather than chemically degraded; continued sonication then fragments these complexes and liberates the bound phenolics, producing the 3 min maximum. The second is analytical, in which the sample volume outside the immediate cavitation zone is under-processed and sampling is correspondingly heterogeneous. The parallel behaviour of total flavonoid content, which also falls at 1 min (0.879 ± 0.045 mg QE/mL against a control of 1.370 ± 0.038 mg QE/mL) before rising at 3 min, indicates that the 1 min depression is a property of the sample and not of a single assay, which favours the first interpretation, the two are not mutually exclusive. The finding on which the conclusions of this study rest, that 3 min sonication elevates extractable phenolics by 33.9% over the untreated control, does not depend on the 1 min value and is corroborated by the parallel 42.3% increase in total flavonoid content at the same duration.

Total flavonoid content (TFC) followed a broadly similar trend. TFC was lowest at US-1 (0.879 ± 0.045 mg QE/mL) and recovered strongly at US-3 (1.949 ± 0.123 mg QE/mL), representing a 42.3% increase over the control (1.370 ± 0.038 mg QE/mL). The values subsequently reduced to 1.414 ± 0.465 mg QE/mL for ultrasonicated samples of 5 min (US-5) and to 1.248 ± 0.173 mg QE/mL with the 7 min (US-7) treatment. Flavonoids in cranberries are often glycoside-conjugated in the vacuolar matrix and hence are sensitive to release during acoustic cavitation. The total flavonoid contents increase during the 3 ultrasound treatment suggests the balance among cell wall disruption and phenolic stability [2], while longer ultrasonication results in the decline in the flavonoid aglycones [48].

The total anthocyanin content of the ultrasonicated samples had 35 ± 0.193 mg CGE/L for the control sample, reduced to 30.674 ± 5.685 for US-1, then to 23.801 ± 1.306 for US-3, 19.972 ± 2.553 mg CGE/L for 5 min (US-5) treatment, then a slight rise to 21.308 ± 0.334 mg CGE/L for the 7 min (US-7) ultrasonication samples. The results showed that the degradation occurred within the 5 min of the treatment. Anthocyanins are responsive to the stress from acoustic oxidation, since their flavylium cation chromophores are attacked by hydroxyl radicals that are produced in transient cavitation collapse [49], [50].

The DPPH radical scavenging activity also declined among all the ultrasonicated samples, from 32.530% for the control to 12.959% at 1 min (US-1) of treatment, with a minimal recovery with the 7 min (US-7) treatment reflecting 23.991%. The pattern with the decline of DPPH despite the rise of total phenolic contents at 3–––7 min of treatment suggests a shift in the phenolic compound pool, that is, the released phenolics, responsible for the total phenolics rises, may possess a lower per-unit DPPH scavenging capability than the anthocyanins, flavonoids, and procyanidins. FRAP values reduced with the increase in ultrasonication times, from 0.566 ± 0.070 mmol TE/mL for the control to 0.244 ± 0.009 mmol TE/mL at 7 min (US-7) of treatment, which reflects a 56.9% decline. The progressive FRAP reduction parallels the monotonous total anthocyanin loss, coherent with strong association between the anthocyanin content and ferric-reducing antioxidant potential in cranberry juice [3], [4].

The divergence between rising total phenolic content and falling radical-scavenging capacity is quantitatively substantial here, a 33.9% TPC gain at 3 min against a 60.2% DPPH loss at 1 min and a 56.9% FRAP loss at 7 min, and it is larger than the divergence reported in comparable studies. [35], reviewing ultrasound processing of fruit and vegetable juices, report antioxidant capacity to be broadly retained or modestly increased under short sonication, and [34]report concurrent increases in both phenolics and antioxidant activity in phalsa juice. The present result differs from both, and the difference is explicable by the composition of the cranberry matrix rather than by processing distortions. The antioxidant capacity of cranberry juice is dominated not by free phenolic acids but by anthocyanins and by oligomeric proanthocyanidins, both of which carry multiple *ortho*-dihydroxy donor sites per molecule and are disproportionately effective per unit mass in the DPPH and FRAP assays. Sonication depletes precisely this fraction, with total anthocyanins falling 43.0% by 5 min and procyanidin B2 falling 20.2% by 7 min, while the phenolic species it releases are predominantly lower-molecular-weight cell-wall-bound acids with fewer donor sites per unit mass. In juices whose antioxidant capacity resides in the free phenolic pool rather than in an oligomeric fraction, no such divergence would be expected, which is why it is not generally reported in the literature.

### 3.3 Vitamin C and carotenoids analysis of ultrasound treated cranberry juice

The effects of ultrasonication on vitamin C (ascorbic acid) and total carotenoid content of cranberry juice are presented in Table 2. These two micronutrient classes showed contrasting responses to acoustic cavitation, reflecting their differing structural stabilities and localization within the cranberry juice matrix. Statistical grouping letters (Tukey HSD, p < 0.05) are included in the table.

**Table 2:** Effect of ultrasound treatment on vitamin C and carotenoid content of cranberry juice.

Sr no.	Sample	Vitamin C	Carotenoids
1	Untreated	2.445 ± 0.114^a^	0.873 ± 0.146^b^
2	US-1	2.259 ± 0.031a^b^	0.821 ± 0.199^b^
3	US-3	2.219 ± 0.185a^b^	0.935 ± 0.166^b^
4	US-5	1.632 ± 0.206^c^	1.752 ± 0.435^a^
5	US-7	2.157 ± 0.209^b^	1.891 ± 0.676^a^

The vitamin C and carotenoid contents of ultrasonicated cranberry juice are presented in Table 2. Ascorbic acid contents showed a progressive decrease with treatment time increase from 2.445 ± 0.114 mg/L for the control to 2.259 ± 0.031 mg/L at 1 min (US-1), representing a 7.6% decrease, and 2.219 ± 0.185 mg/L at US-3 (9.2% decrease) with a pronounced loss at US-5, with1.632 ± 0.206 mg/L (33.2% decrease) followed by partial recovery at US-7 with 2.157 ± 0.209 mg/L (11.8% decrease).

The ascorbic acid trajectory is non-monotonic, with a pronounced minimum at 5 min (1.632 ± 0.206 mg/L, a 33.2% loss) and a partial return at 7 min (2.157 ± 0.209 mg/L, an 11.8% loss), and this apparent recovery requires careful treatment because ascorbic acid cannot be synthesised during processing. Three explanations are consistent with the data and cannot be separated by the present measurements. First, the assay used is a ferric-reduction spectrophotometric method that responds to reducing capacity rather than to ascorbic acid specifically; phenolic compounds released progressively at longer treatment durations, as evidenced by the elevated total phenolic content at 5 and 7 min, are themselves ferric reductants and can inflate the apparent ascorbic acid value. Second, oxidation of ascorbic acid to dehydroascorbic acid is reversible, and partial reduction back to ascorbic acid by the residual polyphenol pool has been reported in sonicated juices [18], which would produce a real but modest recovery. Third, the difference between the 5 and 7 min means is 0.525 mg/L against replicate standard deviations of 0.206 and 0.209 mg/L, so part of the apparent recovery may be replicate variability. A genuine chemical recovery is therefore not established by these data alone, and confirmation requires HPLC quantification of ascorbic acid and dehydroascorbic acid separately, which is recommended for future work.

Total carotenoid content (TCC) demonstrated a contrasting and progressive increasing pattern with increasing treatment. While US-1 yielded a slight reduction, with 0.821 ± 0.146 μg/mL compared to 0.873 ± 0.146 μg/mL for the control, carotenoid content increased progressively at US-3 (0.935 ± 0.166 μg/mL, +7.2%), US-5 (1.751 ± 0.435 μg/mL, +100.8%), and US-7 (1.891 ± 0.676 μg/mL, +116.9%). The observed increase values with longer treatment times are attributable to cavitation-induced break-down of carotenoid-protein and carotenoid-lipid complexes from food matrixes [51]. The enhancement with 5 and 7 min (US-5 and US-7) of treatment is a reflection that longer durations of ultrasonication could effectively increase the bioaccessibility of lipophilic bioactive compounds in juices, in spite of the decline of water-soluble antioxidant compounds [35].

### 3.4 Impact of ultrasound treatment procyanidin A1 and B2

**Fig. 3:**
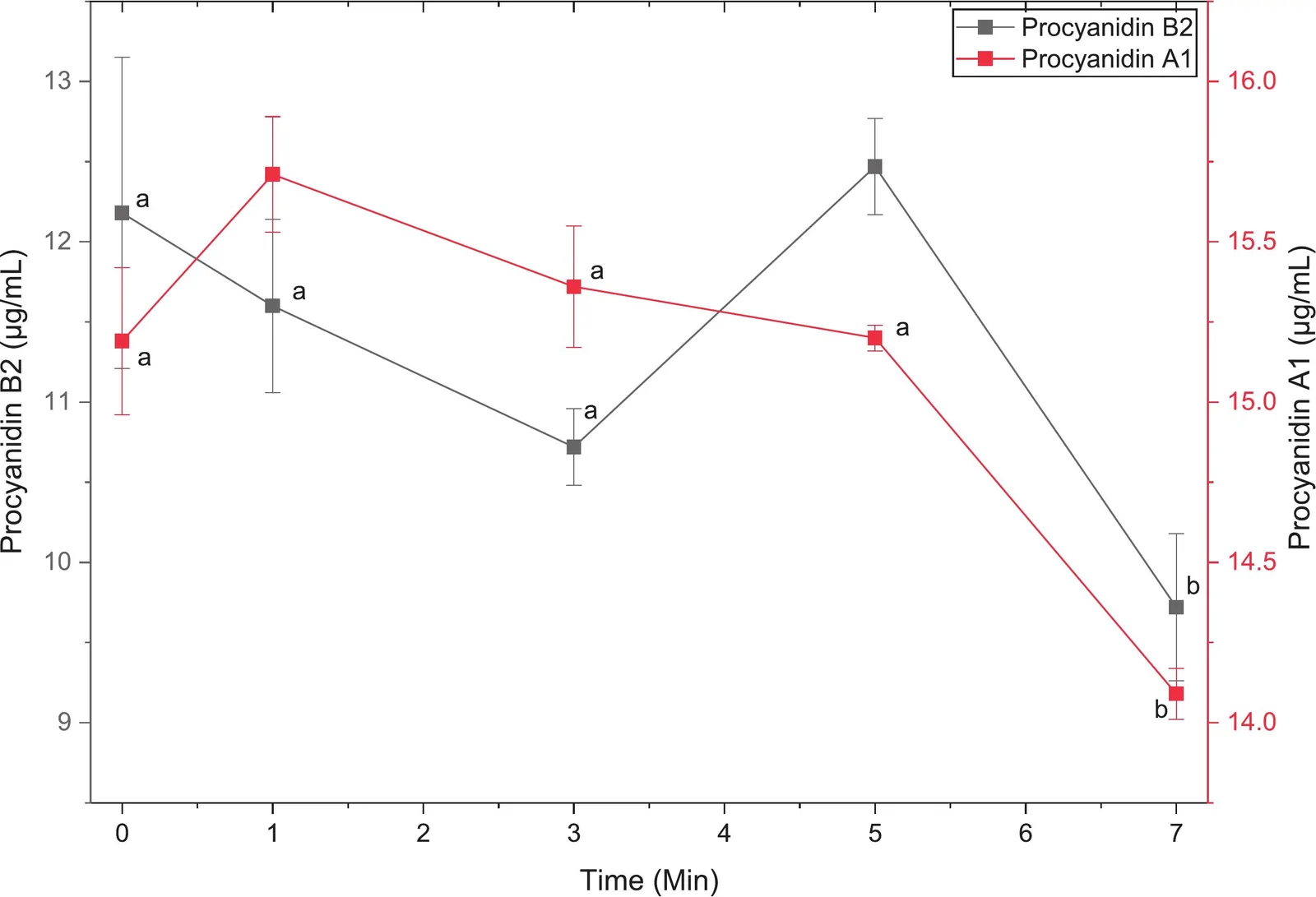
Effect of ultrasound treatment on Procyanidin A1 and B2. Statistical significance is illustrated with a different letter (a or b).

The procyanidin contents of ultrasonicated cranberry juice are presented in Fig. 3. Procyanidin A1, a double-linked A-type flavan-3-ol dimer, demonstrated remarkable stability across most treatment durations. The values recorded were closer to the untreated control sample with 15.19 ± 0.23 μg/mL, ultrasonication of 1 min with 15.71 ± 0.18 μg/mL (+3.4% increase), 3 min with 15.36 ± 0.19 μg/mL (+1.1% increase), and 5 min (US-5) with 15.20 ± 0.04 μg/mL (negligible + 0.1% increase), while a decline was noted at 7 min (US-7) of treatment with 14.09 ± 0.08 μg/mL (−7.2% decrease).

The stable nature of A-type procyanidins is attributable to their double linkages of C-O-C and C-C inter-flavan bonds, which give rigidity and resistance to hydrolytic and oxidative destruction with acoustic shear in comparison to B-type dimers [52], [53].

Procyanidin B2, which is a single linked B-type dimer, had sensitivity to the ultrasonication treatment. Procyanidin B2 concentrations reduced consistently from 12.18 ± 0.97 μg/mL for the control to 10.72 ± 0.24 μg/mL at 3 min (US-3) ultrasonication (a decrease of −11.9%) and then achieved a minimal value at 7 min (US-7) of treatment with 9.72 ± 0.46 μg/mL, representing a 20.2% decrease. There was a retention with the 5 min treatment with 12.47 ± 0.30 μg/mL, which suggests a non-monotonic response, of competing mechanisms that is, acoustic disruption during processing releasing additional B-type oligomers from the cranberry juice matrix by potentially offsetting their simultaneous oxidative degradation at intermediate periods [54].

The differential response between Procyanidin A1 and B2 corroborates the structure-dependent stability of procyanidin subclasses under acoustic stress and has important implications for optimizing ultrasonication parameters to preserve the cranberry-specific A-type procyanidin profile, which confers anti-adhesion activity against uropathogenic bacteria [2].

### 3.5 Impact of ultrasound treatment on minerals content

The mineral composition of ultrasonicated cranberry juice as determined by ICP-OES analysis is presented in Table 3. Minerals are not degraded by acoustic cavitation; rather, ICP-OES-detected concentrations reflect treatment-dependent changes in the extractable/detectable mineral fraction. ICP-OES-detected macro-mineral concentrations were generally lower at short treatment durations relative to the untreated control, with progressive recovery at longer durations.

**Table 3:** Effect of ultrasonication treatment duration on the mineral composition (μg/mL) of cranberry juice as determined by ICP-OES analysis.

Treatment		Macro-minerals					Micro-minerals			
Sr no.	Sample	Ca	Na	K	Mg	P	Zn	Fe	Se	Mn
**1**	Untreated	0.74 ± 0.00^a^	356.20 ± 0.14^ab^	10.45 ± 0.00^a^	0.55 ± 0.01^a^	0.41 ± 0.00^c^	67.45 ± 0.07^a^	137.60 ± 0.14^a^	9.25 ± 0.07^b^	239.20 ± 0.14^b^
**2**	US-1	0.47 ± 0.00^d^	171.55 ± 0.21^d^	8.14 ± 0.00^c^	0.38 ± 0.00^cd^	0.16 ± 0.00^d^	40.25 ± 0.07^b^	81.70 ± 0.14^c^	3.50 ± 0.14^c^	199.15 ± 0.07^d^
**3**	US-3	0.60 ± 0.00^c^	259.20 ± 0.14^c^	8.70 ± 0.00^bc^	0.45 ± 0.14^b^	0.33 ± 0.00^cd^	64.65 ± 1.06^ab^	85.50 ± 0.28^c^	10.90 ± 0.00^a^	229.70 ± 0.14^c^
**4**	US-5	0.63 ± 0.01^bc^	336.20 ± 0.14^b^	9.29 ± 0.01^ab^	0.48 ± 0.00^b^	0.59 ± 0.00^b^	66.80 ± 0.14^a^	129.70 ± 0.14^ab^	1.85 ± 0.07^d^	244.40 ± 0.14^b^
**5**	US-7	0.71 ± 0.00^ab^	385.45 ± 0.07^a^	10.23 ± 0.01^a^	0.40 ± 0.00^bc^	0.82 ± 0.00^a^	66.55 ± 0.21^a^	105.25 ± 0.07^b^	2.50 ± 0.14^cd^	270.45 ± 0.07^a^

Values are means ± SD. Different superscript letters within a column denote significant differences (p < 0.05, one-way ANOVA, Tukey HSD).

The ICP-OES-detected sodium concentration was 171.55 ± 0.21 μg/mL at US-1 (vs. 356.20 ± 0.14 μg/mL for the untreated control, −51.8%), recovering to 259.20 ± 0.14 (US-3), 336.20 ± 0.14 (US-5), and 385.45 ± 0.07 μg/mL (US-7). ICP-OES-detected potassium concentrations similarly showed lower values at US-1 (8.14 μg/mL vs. control 10.45 μg/mL), with recovery at longer durations. Calcium and phosphorus followed comparable patterns. These trends are consistent with cavitation-induced disruption of cranberry cell matrices progressively releasing bound mineral fractions into the detectable juice medium at longer treatment durations [3]. The high Na (385.45) and K (10.23) with the 7 min (US-7) ultrasonicated samples, which is same or exceeds the values of the control reflects accumulated release from disrupted intracellular compartments with the progressive cavitation exposure. The micro-minerals, that is Zn, Fe, Se, and Mn, had an analogous trend, with the 1 min (US-1) of ultrasound treated sample declining and subsequent samples progressively recovering with longer duration of ultrasonication. Zinc reduced from 67.45 μg/mL with the control sample to 40.25 μg/mL at 1 min (US-1) of ultrasonication, with observed recovery to 64–66.80 μg/mL at ultrasound treatment 3–7 (US 3–7) min of treatment.

Iron exhibited a similar pattern, reaching a lowest point at 1 min (US-1) of treatment (81.70 μg/mL) and partially recovering at 5 min (US-5) 129.70 μg/mL before declining slightly at 7 min (US-7) 105.25 μg/mL. The imbalance observed with selenium may be attributed to its minimum quantity as well as its sensitivity to analytic interferences. These findings from the results signify that modest ultrasonication treatment durations from 3 to 5 min (US-3-5) provide favorable compromise, which enable intracellular disruption for polyphenol and mineral extraction and retention while restraining the oxidative decline of water-soluble antioxidants compounds [4].

Copper is not reported in Table 3. Blank-corrected copper concentrations were negative in every sample, including the untreated control, which indicates net emission intensity below that of the reagent blank rather than a physically meaningful concentration. Copper was therefore below the method detection limit under the digestion and dilution conditions used and has been removed from the table. The values that remain are ICP-OES-detectable concentrations on an as-digested basis, and they are intended for comparison between treatments within this study rather than for absolute comparison against compositional tables generated under different digestion and dilution protocols.

### 3.6 Microbial analysis

**Fig. 4:**
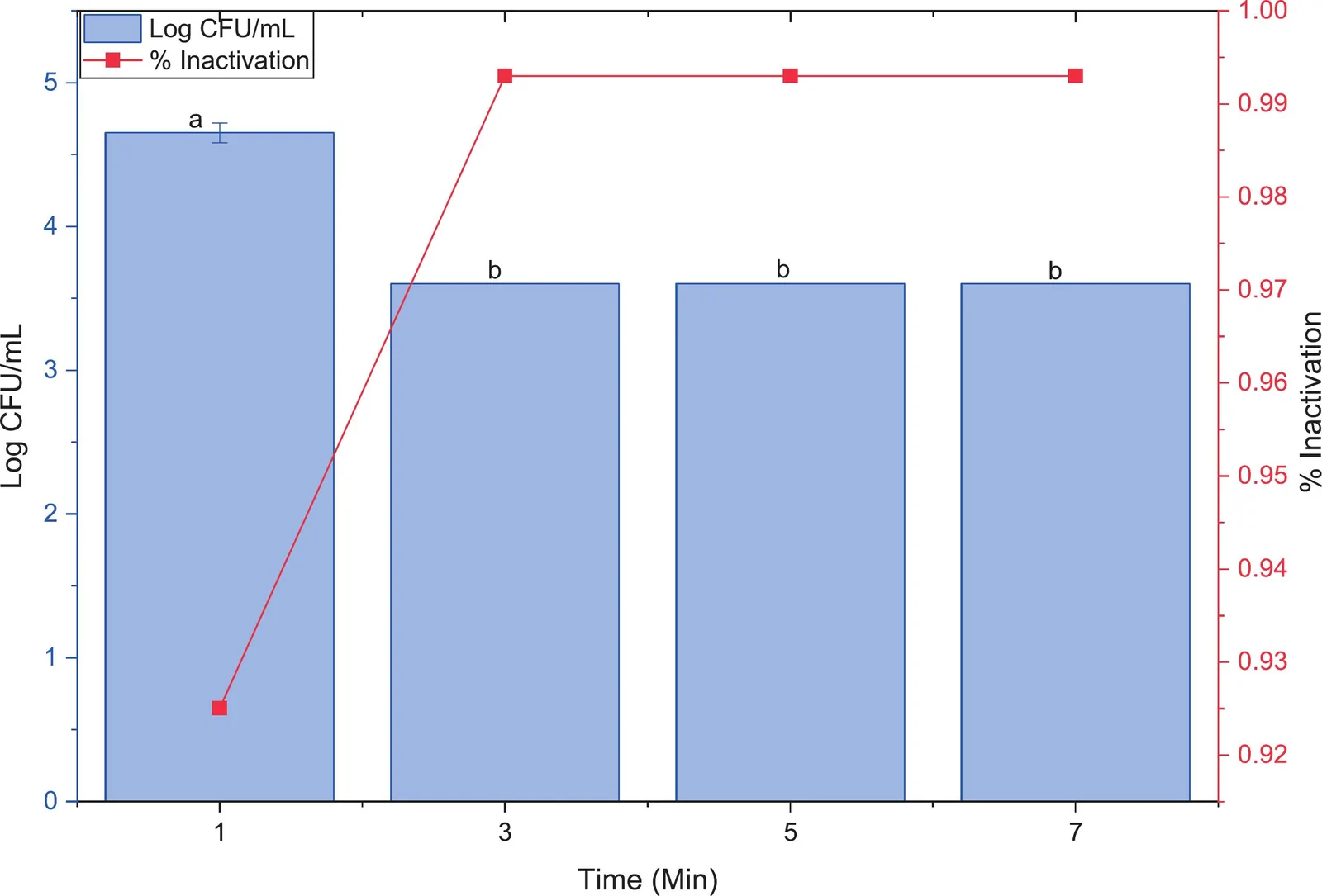
Effect of ultrasonication treatment duration on total plate count (Log CFU/mL) of cranberry juice. Error bars represent ± standard deviation of duplicate measurements. Statistical difference is indicated by different letters.

The effect of ultrasonication on the total plate count (TPC) of cranberry juice is presented in Fig. 4. The untreated control recorded a TPC of 5.602 log CFU/mL. The ultrasonicated cranberry juice samples produced statistically significant decreases in the analysis of the microbial counts relative to the control at p < 0.05. The ultrasound treated cranberry sample for 1 min (US-1) had a reduction of 0.951 log CFU/mL, with a measured TPC of 4.651 ± 0.069 log CFU/mL, corresponding to an 89% decrease in viable microbial counts. Effective microbial inactivation was also observed in the 3 min (US-3) treated samples onwards, with samples 3, 5, and 7 (US3, 5 and 7) producing identical values of 3.602 ± 0.000 log CFU/mL, representing a 2.000 log reduction, hence 99% inactivation, in comparison to the control. The plateau in deactivation from treatment times of 3 to 7 min (US-3 to 7) implies that maximum microbial reduction under the applied dilution conditions was achieved by US-3, with extended ultrasonication offering no additional benefit in total plate count reduction [2].

The inactivation of microorganisms by ultrasonication is attributed primarily to acoustic cavitation, in which the formation and violent collapse of microbubbles generates intense localized pressures (up to 500  MPa) and temperatures (approximately 5000  K), as well as high-velocity micro-jets and shear forces capable of disrupting microbial cell walls and membranes [3]. The transient production of hydroxyl radicals (OH) and hydrogen peroxide (H_2_O_2_) during cavitation collapse further contributes to oxidative damage to microbial DNA, membrane lipids, and structural proteins, impeding cellular repair mechanisms [4]. The sharp decrease in total plate count at US-3 relative to US-1 indicates that a threshold ultrasonication energy is required to shift the dominant inactivation mode from membrane permeabilization to irreversible cell lysis. The findings confirm that ultrasonication at treatment durations of 3 min (US-3) or longer provides effective microbial reduction in cranberry juice, supporting its viability as a non-thermal decontamination strategy that concurrently preserves bioactive compounds at moderate treatment levels.

Two points of the microbiological component is stated explicitly. First, inactivation was assessed against a single inoculated organism, *Escherichia coli K12* (ATCC 15597), a non-pathogenic surrogate selected for its established use as a process indicator and for containment reasons. Gram-negative rods are among the more ultrasound-susceptible vegetative organisms because their thinner peptidoglycan layer offers less resistance to cavitational shear, so the 2.00 log reduction reported here is seen as an upper bound on the performance of this process against the microbial population of concern in juice. The 5 log pathogen reduction required by the United States FDA Juice HACCP rule (21 CFR 120) is not demonstrated by these data and is not claimed. Second, the plateau observed from 3 to 7 min at 3.602 ± 0.000 log CFU/mL, with zero variance across replicates, indicates that the detection floor of the plating and dilution scheme was reached rather than that inactivation ceased; the true reduction at 5 and 7 min may therefore exceed 2.00 log.

No thermal control was run in parallel in this study, so a direct efficacy comparison with conventional pasteurisation cannot be made from these data. The comparison that can be made is against the published literature: conventional HTST pasteurisation of juice at 90 °C for 30 s or 72 degrees C for 15 s routinely achieves greater than 5 log reduction of vegetative pathogens, against the 2.00 log demonstrated here. Ultrasonication as applied in this study is therefore not a substitute for thermal pasteurisation as a standalone critical control point. Its realistic role is either as a pre-treatment permitting a reduced thermal load in a combined process, or in thermosonication and manosonication configurations in which mild heat or elevated pressure is applied concurrently and synergistic inactivation is obtained; thermosonication has been reported to deliver the required log reductions at temperatures well below conventional pasteurisation, with correspondingly better retention of thermolabile compounds [[3], [34]].

### 3.7 Practical considerations, energy demand, scalability and regulatory position

The energy delivered in the 3 min optimum treatment was approximately 27,000 J to a 100 mL sample, equivalent to a specific energy input of approximately 270 kJ L-1, or 0.075 kWh L-1 on a delivered-acoustic basis. Electrical demand at the wall is higher by the electroacoustic conversion efficiency of the transducer, typically 60–80% for a 20 kHz piezoelectric stack, giving an order-of-magnitude figure of 0.09–0.13 kWh L-1. For context, HTST pasteurisation of juice with conventional regenerative heat exchange operates at roughly 0.02–0.05 kWh L-1. Ultrasonication as configured here is therefore not energy-competitive with thermal processing on a per-litre basis, and its industrial case rests on product quality rather than on operating cost.

Scale-up from the batch probe configuration used here non-linear. Acoustic intensity falls steeply with distance from the probe tip, so that in a batch vessel an active cavitation zone of a few cubic centimetres immediately below the horn is coupled to a much larger passive volume, and treatment uniformity depends entirely on convective circulation through that zone. Increasing batch volume at constant power therefore reduces the mean cavitation dose and widens its distribution, and results obtained on 100 mL cannot be extrapolated directly to vessel scale. The industrially realistic configuration is a continuous flow-through cell in which juice is pumped through an annular or radial sonication chamber at a residence time matched to the required energy density, with multiple transducers arranged to eliminate dead zones. Under flow the controlling variable ceases to be treatment time and becomes specific energy input per unit volume, and the figure of 270 kJ L-1 derived above is the transferable design parameter from this study.

No storage trial was included in this study, and no claim about shelf-life extension can be made from it. On economics, the capital cost of a flow-through ultrasonic system is modest relative to a pasteurisation line, but operating energy cost is higher, so the process is most plausibly viable in premium chilled or functional-beverage segments where retention of the A-type procyanidin profile can be substantiated on-label and carries a price premium, rather than in commodity juice production.

Table 4 sets the process demonstrated here against the thermal and non-thermal technologies currently in commercial use for fruit juice, using indicative ranges compiled from the published literature; only the ultrasonication row derives from measurements made in this study. The comparison places the present results in context: ultrasonication is the mildest of the listed technologies in thermal terms and is the only one that actively increases extractable phenolic and flavonoid content, but it is also the lowest in microbial inactivation and, uniquely, imposes a substantial oxidative penalty on the anthocyanin fraction. Its competitive position is therefore not as a general-purpose replacement for pasteurisation but as a bioactive-release step whose microbial contribution is partial but beneficial.

**Table 4:** 

Technology	Typical pathogen log reduction in juice	Bulk process temp.	Reported anthocyanin retention	Indicative energy demand (kWh L-1)	Principal limitation for cranberry juice	References
HTST thermal pasteurization (72–95C, 15–30 s)	> 5 log	72–95 °C	60–80%	0.02–0.05	Degrades thermolabile anthocyanins and ascorbic acid; cooked flavor note; no bioactive release benefit	[55]
High-pressure processing (400–600 MPa, 3–5 min)	> 5 log	< 40 °C	85–95%	0.10–0.20	Batch operation; high capital cost; ineffective against bacterial spores	[56]
Pulsed electric field (20–40 kV cm-1)	4–6 log	< 45 °C	80–95%	0.03–0.10	Requires low product conductivity; electrode fouling; ineffective against spores	[57]
UV-C irradiation (254 nm)	3–5 log	Ambient	> 90%	0.01–0.03	Requires high UV transmittance; poorly suited to a deeply pigmented, turbid juice	[58]
Thermosonication (20 kHz + 50–60C)	> 5 log	50–60 °C	70–90%	0.06–0.12	Reintroduces a partial thermal load; process validation more complex	[59]
Ultrasonication, this study (20 kHz, 3 min, ∼270 kJ L-1)	2.00 log (censored at detection floor)	Not exceeding 25 °C	57% retained at 5 min (43% loss)	0.09–0.13 (estimated)	Insufficient alone for 5 log compliance; oxidative loss of anthocyanins, ascorbic acid and B-type procyanidins	This study

Table 4 has indicative comparison of the ultrasonication process demonstrated in this study with thermal and non-thermal technologies in commercial use for fruit juice processing. Values for all technologies other than ultrasonication are indicative ranges compiled from the published literature for orientation and were not measured in this study; energy figures are order-of-magnitude estimates that vary substantially with plant configuration and through-put.

## 4 Conclusion

This study establishes the treatment-duration window within which 20 kHz probe ultrasonication improves the extractable bioactive profile of cranberry juice without compromising the structural fraction that carries its distinctive functionality. The principal scientific contribution is the demonstration that A-type and B-type procyanidins diverge under identical acoustic conditions: procyanidin A1 remained within 3.4% of the untreated control at every duration up to 5 min, and within 1.1% at 3 and 5 min, while procyanidin B2 fell to 20.2% below the control at 7 min, establishing that the doubly-linked A-type inter-flavan bond confers resistance to cavitational and oxidative stress, and that the cranberry-specific anti-adhesion fraction can be carried through a sonication process that simultaneously achieves substantial microbial reduction. Treatment for 3 min emerged as the optimum, delivering a 33.9% increase in total phenolic content, a 42.3% increase in total flavonoid content, a 2.00 log reduction in inoculated *E. coli K12* and negligible change in procyanidin A1, with total soluble solids, pH and colour essentially preserved (total colour difference not exceeding 1.82).

These gains were obtained with cost, and the trade-offs were that, the total anthocyanin content fell by 43.0% at 5 min, ferric-reducing antioxidant power by 56.9% at 7 min, and ascorbic acid by 33.2% at 5 min. Ultrasonication of cranberry juice is therefore an exchange of the oxidisable pigment and vitamin fraction for improved accessibility of the phenolic, flavonoid and carotenoid fractions, and its value in any given application depends on which of these attributes the product is formulated to deliver.

No storage trial and no sensory evaluation were conducted, so neither shelf-life nor consumer-acceptability claims are made. All results were obtained at 100 mL batch scale in a single fixed probe geometry.

Future work should look into examining a parallel thermal control and a thermosonication to position the process against conventional pasteurisation and to establish whether the required log reductions can be reached at a thermal load low enough to preserve the quality advantages reported here. Also, a continuous flow-through configuration at a constant specific energy input of approximately 270 kJ L-1, coupled to a refrigerated storage trial and a techno-economic assessment against a thermal baseline, is required before industrial implementation can be evaluated. Subject to these qualifications, ultrasonication remains a promising green processing route for cranberry juice, most essentially, as a component of a combined process rather than as a standalone replacement for thermal pasteurisation.

## CRediT authorship contribution statement

**Mabel Kyei Kwofie:** Writing – original draft, Visualization, Methodology, Investigation, Formal analysis, Conceptualization. **Valerie Orsat:** Writing – review & editing, Supervision, Funding acquisition.

## Declaration of competing interest

The authors declare that they have no known competing financial interests or personal relationships that could have appeared to influence the work reported in this paper.
